# Supplementary material for: Rescue of Methionine Dependence by Cobalamin in a Human Colorectal Cancer Cell Line
Source: Nutrients. 2024 Mar 28;16(7):997. doi: 10.3390/nu16070997 (PMC11013648; doi:10.3390/nu16070997)
Supplement: Supplementary file 1 [file nutrients-16-00997-s001.zip › nutrients-2912881-supplementary.pdf]

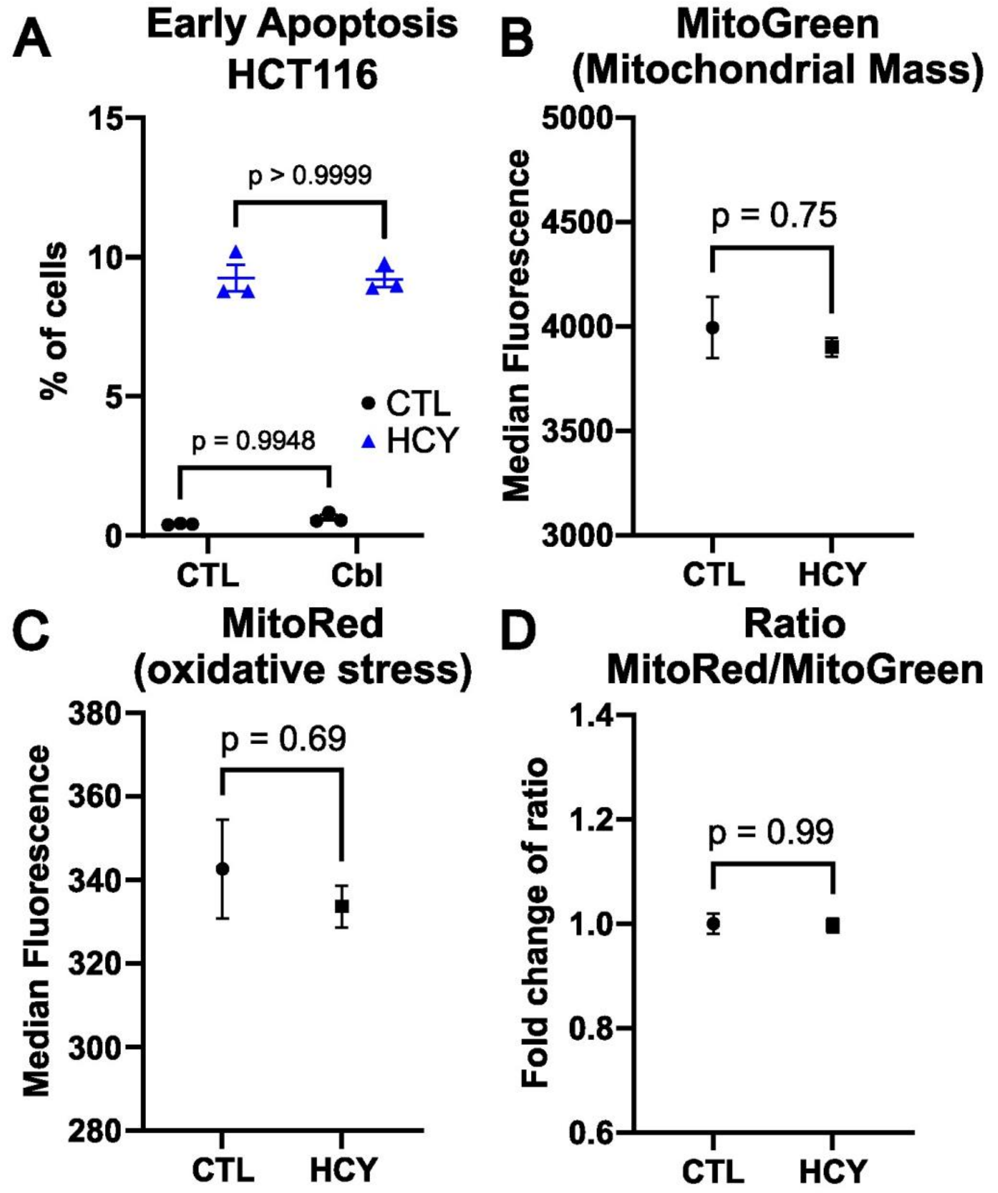

Supplementary Figure S1. Apoptosis and oxidative stress. Cobalamin does not influence early apoptosis (Annexin V positive, PI negative cells) in non-responsive HCT116 cells (A). Oxidative stress does not change in SW48 cells grown in methionine versus homocystine. Flow cytometry measurement of MitoGreen (B), MitoRed (C) and the ratio of MitoGreen to MitoRed (D). CTL: Control, Cbl: cobalamin, HCY: homocystine. Two-way ANOVA with Šídák's multiple comparison test, standard error of mean and 2 of the 6 comparisons shown (A). Unpaired t test, standard error of mean (B-D).

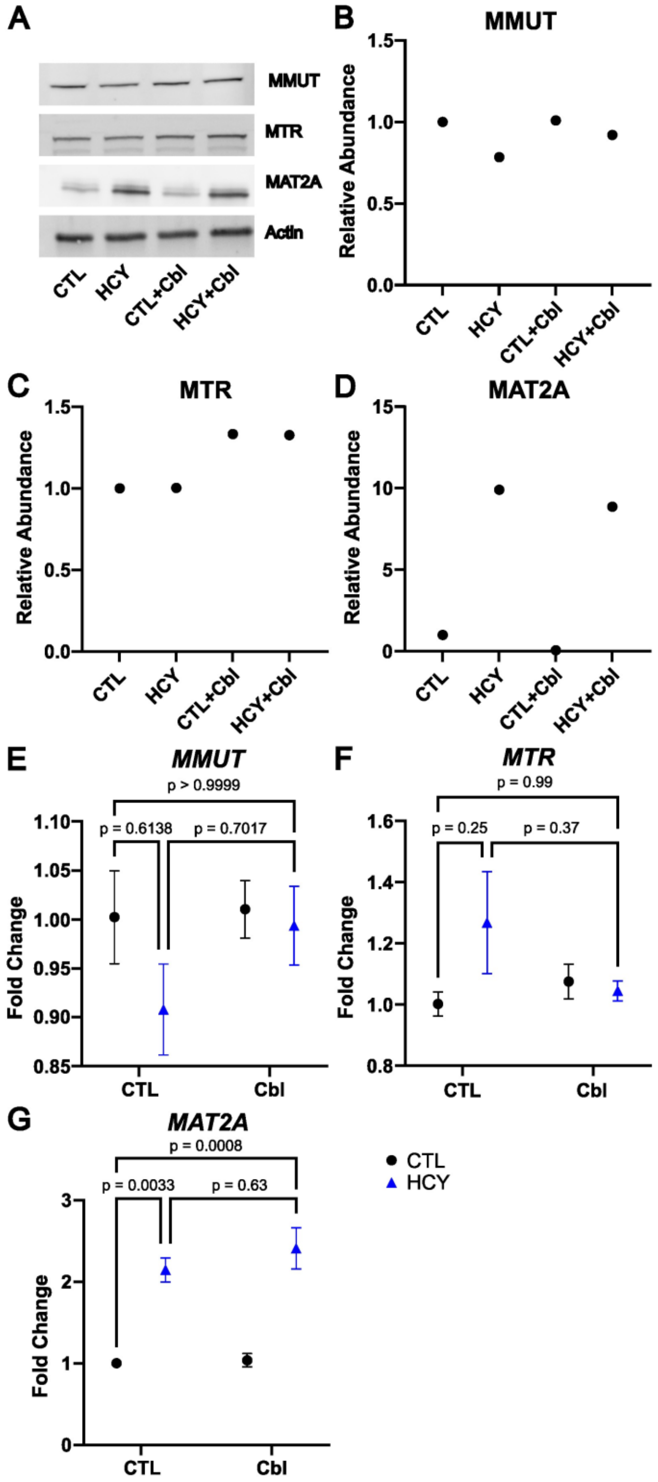

Supplementary Figure S2. Protein abundance and gene expression in SW48 with treatment. Western blot of the proteins MMUT, MTR, and MAT2A compared to the loading control β-actin (A). Quantified protein abundance relative to β-actin for MMUT (B), MTR (C), and MAT2A (D). Gene expression of the genes *MMUT* (E), *MTR* (F), and *MAT2A* (G). CTL: Control, Cbl: cobalamin, HCY: homocystine (Blue). Two-way ANOVA with Šídák's multiple comparison test, standard error of mean and 3 of the 6 comparisons shown.
